# Application of Flow Field Analysis in Ion Beam Figuring for Ultra-Smooth Machining of Monocrystalline Silicon Mirror

**DOI:** 10.3390/mi13020318

**Published:** 2022-02-18

**Authors:** Zhe Wang, Lingqi Wu, Yuanyuan Fang, Aihuan Dun, Jiaoling Zhao, Xueke Xu, Xiaolei Zhu

**Affiliations:** 1 Shanghai Institute of Optics and Fine Mechanics, Chinese Academy of Sciences, Shanghai 201800, China; wangzhe@siom.ac.cn (Z.W.); fangyuanyuan@siom.ac.cn (Y.F.); dunaihuan0810@163.com (A.D.); jolin923@siom.ac.cn (J.Z.); xuxk@siom.ac.cn (X.X.); xlzhu@siom.ac.cn (X.Z.); 2 Center of Materials Science and Optoelectronics Engineering, University of Chinese Academy of Sciences, Beijing 100049, China

**Keywords:** ion beam figuring, monocrystalline silicon mirror, flow field analysis, ultra-smooth surface

## Abstract

X-ray free-electron lasers are large modern scientific devices that play an important role in fields such as frontier physics and biomedicine. In this study, a light source is connected to an experimental station through beam lines, which requires numerous ultra-smooth and high-precision X-ray mirrors. Monocrystalline silicon is an ideal substrate material where ion-beam figuring is required. However, the ultra-smooth surface is damaged after the ion-beam figuring. Through an analysis of the machined surface, it is found that in the process of vacuum pumping, the impurities in the cavity adhere to the machined surface and increase the roughness after processing. Therefore, an optimized vacuum-pumping scheme is proposed. The experiment demonstrates that the original value of the processed surface roughness remains unchanged.

## 1. Introduction

X-ray free electron laser (XFEL) devices have the advantages of bright intensity, high collimation, high brightness, narrow pulse, high polarization, and wide and continuously adjustable energy, which are unmatched by many conventional and laboratory light sources [1,2,3,4]. Brightness is a key indicator of X-ray light sources. Higher brightness means that X-ray detection can obtain a higher space–time resolution in equal dimensions of energy [5,6,7]. Therefore, XFEL is acclaimed as the brightest light. It is an irreplaceable major scientific device for multi-disciplinary frontier research, which has been highly valued by various countries. The beam line is tens to hundreds of meters long, and many optical elements are required to realize modulation processes, such as monochrome, collimation, deflection and focusing [8,9,10]. Most X-ray optical elements are grazing incidence mirrors. Owing to the short wavelengths, there are high requirements for surface quality and morphology. Monocrystalline silicon materials exhibit strong mechanical and thermal properties. The lattice is arranged neatly and densely. After chemical mechanical polishing (CMP), sub-nano surface roughness can be obtained. Thus, it is an ideal material for manufacturing X-ray mirrors.

Ion beam figuring (IBF) has the advantages of high certainty [11,12,13], high-precision, no edge effect and non-contact processing. IBF has become an indispensable processing technology for ultra-precision optical element manufacturing [14,15]. It often undertakes the final finishing task in high-precision surface-shape processing. It is one of the best processes to realize high-precision X-ray mirror shaping. Therefore, IBF is required to maintain the roughness after processing.

The surface roughness of an X-ray mirror has a crucial influence on the reflectivity of coated elements. Therefore, the root mean square (RMS) value of the surface roughness of elements before coating is required to be less than 0.36 nm [16], and after IBF, the surface roughness of the element cannot be reduced. Before the IBF machine works, the vacuum cavity needs to be pumped to 10^−3^ Pa to make the ion source work and produce the ion beam, so vacuum pumping process is a necessary link. At the beginning of vacuum pumping, the air flow in the vacuum cavity quickly forms turbulence, which brings the particle impurities in the vacuum cavity to the element surface. After ion beam bombardment, they are sintered on the element surface, which is difficult to remove and will increase the surface roughness after polishing.

In this paper, aiming at the disadvantage of increasing roughness after machining in the IBF vacuum pumping process, an optimized vacuum pumping scheme to improve roughness is proposed. This technology is analyzed according to theoretical simulation and verified by practical machining experiments.

## 2. Theoretical Analysis

At the beginning of vacuum pumping, the air flow in the vacuum chamber will change dramatically, which will drive the disordered movement of impurity particles in the chamber. This chapter mainly studies the change law of air flow in the chamber. The process of vacuum pumping before IBF is an air flow problem. As air flows from inside the cavity to the outside of the cavity, the flow problem can be described by Navier–Stokes Equations (N-S Equations), as shown in Equation (1)

(1) $$\rho \frac{DV}{Dt}=\rho f-\nabla p+\mu \nabla^{2}V,$$

where *ρ* is the density, *V* is the velocity vector, *P* is the pressure, *f* is the external force per unit volume of the fluid, and *f* = *ρ*g. *μ* is the dynamic viscosity and is a constant. *DV*/*Dt* is a derivative of matter. For any physical quantity *A*, there is

(2) $$\frac{DA}{Dt}=\frac{\partial A}{\partial t}+\left(V\cdot \nabla \right)A,$$

Therefore, the expansion form of N-S equations is

(3) $$\frac{\partial V}{\partial t}+\left(V\cdot \nabla \right)V=f-\frac{1}{\rho }\nabla p+\frac{\mu }{\rho }\nabla^{2}V,$$

For the N-S equation, it is necessary to simplify the calculation. First, the Reynolds number (Re) is calculated using Equation (4):

(4) Re=ρudμ,

where *d* is the characteristic length, *u* is the velocity, and *μ* is the viscosity coefficient of the fluid. Take *ρ* = 1.29 kg/m^3^, *u* = 0.26 m/s, *d* = 0.2 m, and *μ* = 17.9 × 10^−6^ pa·s. Using Equation (4), Re = 3747 > 2000 is calculated, indicating that the air flow in the vacuum pumping process is turbulent.

The Knudsen number (*Kn*) is used to determine whether the fluid is continuous. The calculation formula for *Kn* is

(5) Kn=λL,

where *λ* is the mean-free path (average distance of free movement of molecules between two consecutive collisions), and *L* is the characteristic length. The calculation formula for the mean-free path (*λ*) is

(6) $$\lambda =\frac{1}{\sqrt{2}\pi d^{2}n},$$

where *d* = 0.4 nm for the molecular diameter and *n* = 2.43 × 10^−25^m^−3^ for the gas molecular density. The mean-free path *λ* can be estimated by bringing *d* and *n* into Equation (6). When *Kn* = 3 × 10^−8^ approaches zero in Equation (5), it can be seen that the fluid is continuous. The vacuum-pumping process is a continuous turbulent flow with an outlet and no inlet, which will produce fluid compression. A compressible Euler equation is used to solve [17,18]. Ignoring the fluid viscosity, the N-S equations can be rewritten in the form of the Euler equation:

(7) $$\rho \frac{dV}{dt}=\rho F-\nabla p,$$

The compressible Euler Equation can be expressed as

(8) $$\rho_{t}+{\left(\rho u\right)}_{x}=0,$$

(9) $${\left(\rho u\right)}_{t}+{\left(\rho u^{2}+p\right)}_{x}=0,$$

(10) $${\left(\rho \left(e+\frac{u^{2}}{2}\right)\right)}_{t}+(\rho u\left(e+\frac{u^{2}}{2}\right)+up{)}_{x}=0,$$

where *ρ* is the density, *u* is the velocity, *P* is the pressure, and *E* is the internal pressure. The flow state of the gas in the vacuum cavity can be simulated according to the compressible Euler equation.

**Figure 1:**
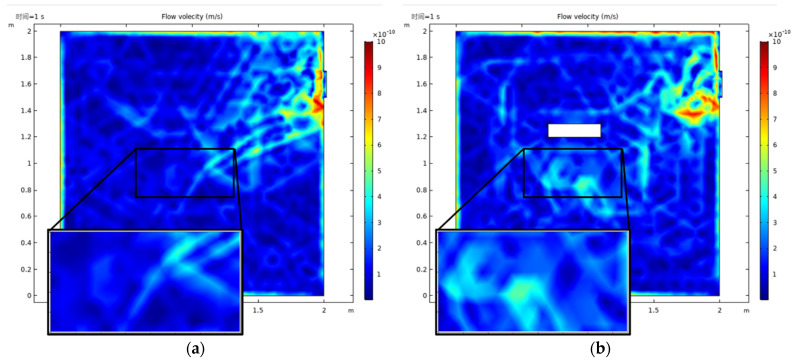
Simulation of vacuum pumping moment without monocrystalline silicon element (**a**) and installation (**b**).

According to the simulation model, the vacuum pumping of an IBF machine without elements in cavity was simulated. The following can be seen in Figure 1a: in the absence of any elements and at the moment air extraction began (*t* = 1 s), the air flow velocity in the cavity changed abruptly; the velocity was fastest at the extraction port; the velocity decreased to the position away from the extraction port; and the gas in the cavity was extracted according to a certain direction and rate. When the elements were installed in the vacuum cavity, the simple and orderly internal structure in the chamber was broken, resulting in a more disordered air flow, as shown in Figure 1b. The white rectangle in the figure represent the elements; the difference in velocity can be clearly seen in the local enlarged view of the effective area in Figure 1.

**Figure 2:**
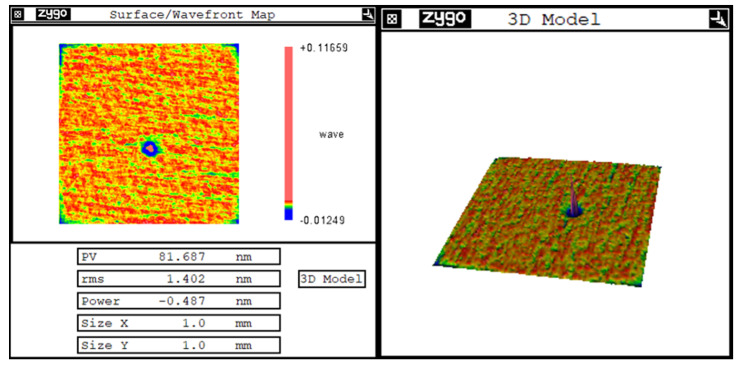
The star-point defect.

For the vacuum system of the conventional IBF machine, the internal structure of the suction port has not been scientifically analyzed, but designed according to the convenience of installation. Therefore, it is usually directly connected to the vacuum cavity at the upper position in the middle of the machine. At the beginning of the vacuum pumping process, the air flow on the lower surface of the monocrystalline silicon mirror moved violently to form turbulence, which drove the particle impurities in the cavity to adhere to the processed surface. After high-temperature bombardment by the ion beam, they were sintered and fixed on the surface of the mirror, which could not be removed, forming star-point defects and reducing the surface roughness of the mirror. Using a white light interferometer, it could be observed that the star-point defect was a bulge, as shown in Figure 2. The transverse dimension was approximately of the order of one hundred microns, and the height was approximately an order of one hundred nanometers. Such star-point defects are difficult to remove when they are attached to the surface of monocrystalline silicon; this affected the surface roughness of the monocrystalline silicon elements, thereby reducing the surface reflectivity after coating and posing the risk of film removal.

**Figure 3:**
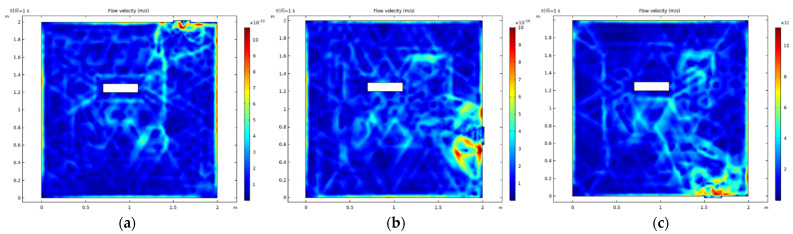
Velocity results at three pumping positions (up (**a**), side down (**b**), down (**c**) position).

**Figure 4:**
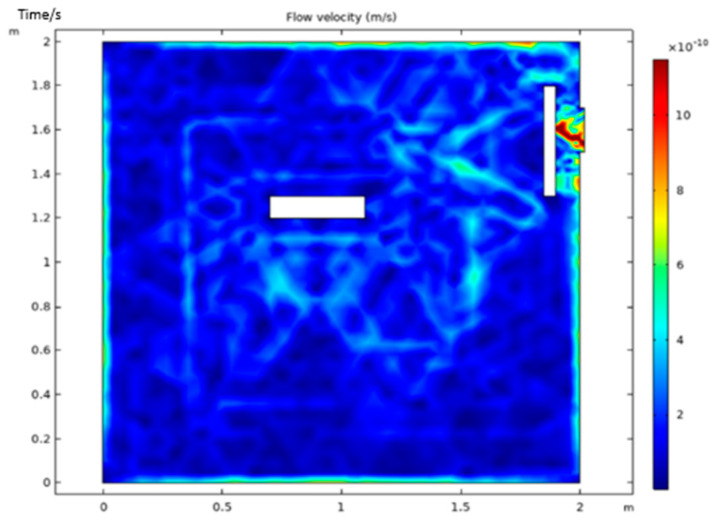
Velocity distribution in the vacuum cavity after installing the baffle.

**Figure 5:**
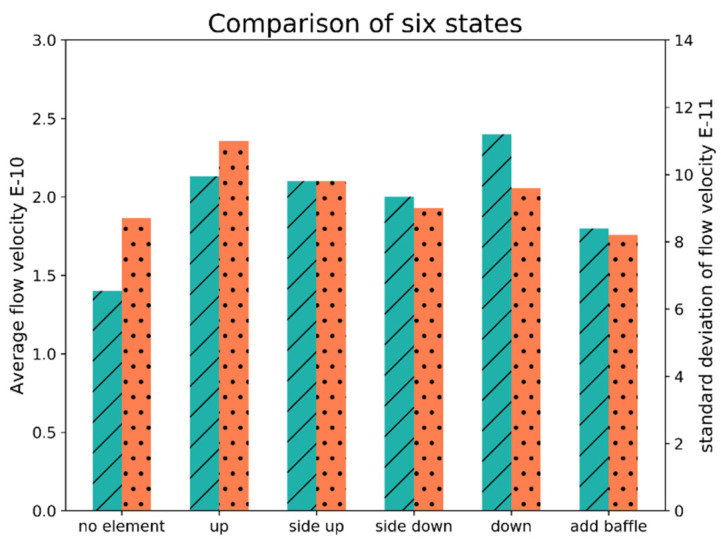
Change in flow velocity under six states; the green stripes represent average flow velocity, and the orange dot represent standard deviation of flow velocity.

As shown in Figure 3, the position of the extraction port of the vacuum cavity was adjusted so that it was in the up position (Figure 3a), side down position (Figure 3b) and down position (Figure 3c). The baffle was added at the extraction port position to allow the elements to be processed and the baffle to divide the cavity into several regular spaces, which would change the velocity of air flow in the cavity, as shown in Figure 4. To obtain the statistics on the velocity distribution in the fixed area of the cavity, the effective area near the lower surface of the element (x (0.6,1.4), y (0.7,1.1)) was selected without installing the element, alongside four different extraction positions. Then, the baffle was installed, and the mean value and standard deviation of the flow velocity were calculated, as shown in Figure 5.

It can be concluded from Figure 5 that when no elements were installed, the average velocity was the smallest and the standard deviation of the velocity was small, which indicated that the velocity change was not abrupt. When the elements were installed, the different pumping positions contributed equally to the degree of disorder of the air flow in the cavity and did not affect the velocity of the air flow in the cavity. With the element installed, the spatial structure distribution in the cavity changed, and the air velocity and flow stability in the cavity also changed. The side down position had the lowest pumping velocity and the most stable air flow. However, it was still greater than that in the flow state when an element was not installed. As the machine had already been manufactured and the extraction position could not be changed easily, a baffle was added to reduce the disorder of air flow in the cavity. After the baffle was added, the average velocity was further reduced, and the standard deviation was reduced to a minimum value, indicating that the flow rate was slow, and it gradually reached the optimal state.

## 3. Experimental Verification

**Figure 6:**
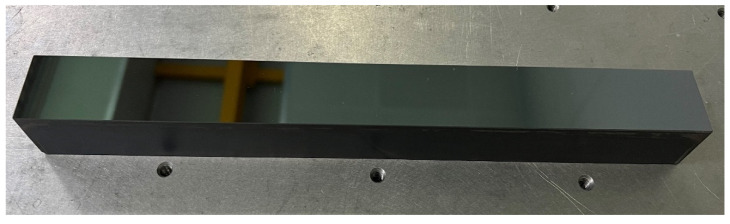
Monocrystalline silicon substrate.

**Figure 7:**
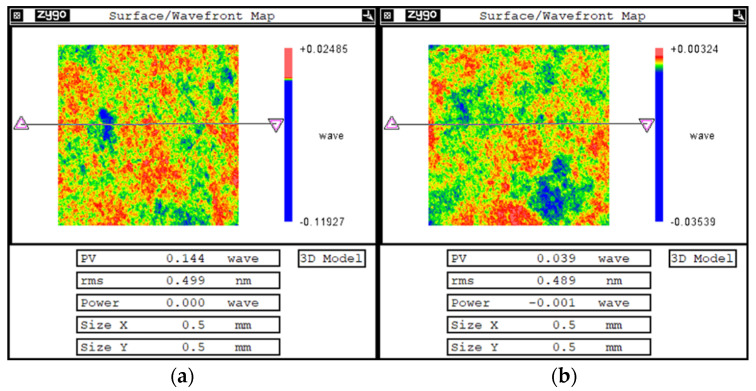
Comparison of roughness before (**a**) and after (**b**) IBF processing.

As shown in Figure 6, a 300 mm monocrystalline silicon strip mirror was installed in the IBF machine. A baffle was added to block the extraction port and reduce the flow rate. After 120 min of IBF processing, the surface quality of the elements and star-point defects were not observed. Surface roughness was measured using a white-light interferometer. Figure 7 shows a comparison of the surface roughness before and after the IBF processing.

It was found that the RMS of surface roughness did not change significantly. Before processing, the RMS of surface roughness was 0.5 nm, and after IBF processing, the RMS was still 0.5 nm, so it did not change. It can be proven that the improved IBF process maintains the surface roughness of the original substrate.

## 4. Conclusions

In this paper, a practical method to maintain the surface roughness after IBF is proposed. By changing the flow field distribution in the vacuum cavity, the air in the cavity is extracted smoothly and orderly, the probability of dust adhering to the element processing surface is reduced, the surface roughness is maintained, and ultra-smooth processing is realized. The feasibility of this method was verified by processing experimental parts. The surface roughness did not change after IBF, which provides strong support for the processing of an X-ray mirror. This technology can also be extended to the processing of other elements.
